# Sirtuin 7 Promotes Mesenchymal to Epithelial Transition by β-Catenin Redistribution and Stabilization

**DOI:** 10.3389/fonc.2020.00740

**Published:** 2020-06-23

**Authors:** Shashi Kiran, Manjari Kiran, Gayatri Ramakrishna

**Affiliations:** ^1^Department of Biochemistry, University of Hyderabad, Hyderabad, India; ^2^Centre for DNA Fingerprinting and Diagnostics, Hyderabad, India; ^3^Department of Systems & Computational Biology, University of Hyderabad, Hyderabad, India; ^4^Department of Molecular and Cellular Medicine, Institute of Liver and Biliary Sciences, New Delhi, India

**Keywords:** SIRT7, MET, β-catenin, Snail, cadherin

## Abstract

SIRT7 belongs to the family of “NAD+ dependent deacetylases” called Sirtuins. In the present work we report a novel role of SIRT7 in regulating cellular polarity. SIRT7 overexpression in immortalized mouse fibroblasts (NIH3T3) induced epithelial transition. This transition was accompanied by typical N- to E- cadherin transition, stabilization of β-catenin, and the downregulation of transcription factors responsible for maintenance of mesenchymal phenotype (Snail, Slug, and Zeb1). Interestingly, a subpopulation of cells overexpressing SIRT7 exhibited an intermediate stage between mesenchymal and epithelial characters. Transformed epithelial cells showed a loss of heterochromatisation as evidenced by a loss of HP1α and H3K9 dimethylation staining. In conclusion, we report a role of SIRT7 in mesenchymal cells, which may have implications for health and disease.

## Introduction

Sirtuins are a family of “NAD+ dependent deacetylases” with diverse cellular and metabolic roles (1, 2). It consists of seven different proteins (SIRT1-SIRT7) localized to different cellular compartments (3). Sirtuin 7 is a nucleolar Sirtuin with diverse roles in rDNA transcription, rRNA processing, metabolic homeostasis, tumorigenesis, chromatin modification, and genomic integrity (4, 5). SIRT7 regulates rDNA transcription through deacetylation of PAF53 and U3-55k, which are components of RNA polymerase-I and U3 snoRNP complexes, respectively (6–8). SIRT7 induces compaction of chromatin through H3K122 desuccinylation and H3K18 deacetylation (4, 9). SIRT7 is also involved in maintenance of metabolic homeostasis by maintaining healthy liver metabolism and cellular energetics through GABP-β1deacetylation (10, 11). Previously, we had identified localization signals for nucleolar targeting of SIRT7 and reported its role in ameliorating DNA damage (12, 13). In the present study we demonstrate a role of Sirtuin 7 in cellular plasticity by inducing epithelial transition in mesenchymal cells.

Role of other Sirtuins in cellular plasticity have been reported (14). SIRT1 overexpression leads to decreased TGF-β, a key cytokine responsible for epithelial to mesenchymal transition (EMT) in mammary cancer cells, thereby decreasing the metastatic ability of these cells (15). SIRT1 impedes metastasis in ovarian cancer by decreasing EMT related changes (16). Contrary to these reports in prostate cancer, SIRT1 acts as a positive regulator of metastasis by accelerating EMT through the transcription factor Zeb1 (17). SIRT2 overexpression accelerates changes associated with epithelial to mesenchymal transition, and its knockdown leads to regression of EMT related changes (18).

Cellular transition between epithelial and mesenchymal types is well-known in many biological processes such as embryogenesis, organ development, and neoplastic transformations (19–21). Transition to alternative cellular fates involves reprogramming, phenotypic changes, and alterations in gene expression. It involves biochemical processes governed by several transcription factors (Snail, Slug, Zeb1, Twist1) to regulate reorganization of cytoskeletal proteins, gap junctions and the extracellular matrix (ECM) (22–24). The most well-studied cellular fate transitions include the epithelial/ endothelial cells to mesenchymal state (EMT). However, relatively little is known about the process of mesenchymal to epithelial cell transition (MET) (25). In the present work we report a role of Sirtuin 7 in the process of MET.

Reports on SIRT7 have demonstrated the contrasting effect of SIRT7 on cellular transition, depending on the origin of cells studied. SIRT7 was found to suppress EMT in oral squamous cells, carcinoma cells, and breast cancer cells by promoting SMAD4 deacetylation, leading to increase of E-cadherin and down regulation of N-cadherin and Vimentin (26, 27). In contrast, SIRT7 promoted EMT in prostate and colorectal cancer cells by suppressing E-cadherin expression (28–30). However, the overall role of SIRT7 in cellular plasticity was not demonstrated and a decrease in tumor size after SIRT7 knockdown was not very appreciable.

In the present study we characterize the role of SIRT7 in mesenchymal to epithelial transition (MET) when overexpressed in fibroblast cells. We observed a morphological transformation accompanied by a change of molecular determinants of cellular plasticity associated with SIRT7 overexpression. We report that SIRT7 induces a cadherin switch from N- to E- type, membrane stabilization of beta-catenin, and down regulation of transcription factors Snail, Slug, and Zeb1.

## Materials and Methods

### Establishment of Stable Cell Lines

NIH3T3 cells overexpressing GFP alone or SIRT7GFP were generated by retroviral transduction. For this cDNA encoding, GFP alone or SIRT7GFP were cloned in pCX4Neo vector and co-transfected with plasmids encoding retroviral VSVG (expressing env) and VSV-GP (expressing gag and pol) in a packaging cell line, PlatE, using Lipofectamine2000 (Invitrogen, USA). Retroviral particles obtained from these cells were used for transducing target NIH3T3 cells. Infected cells were selected in Geniticin for 10 days followed by dilution plating in 96 well plates. Stable lines were established by expanding the colonies after dilution plating.

### Isolation of Two Morphologically Distinct SIRT7 Overexpressing Cell Lines

**Figure 1:**
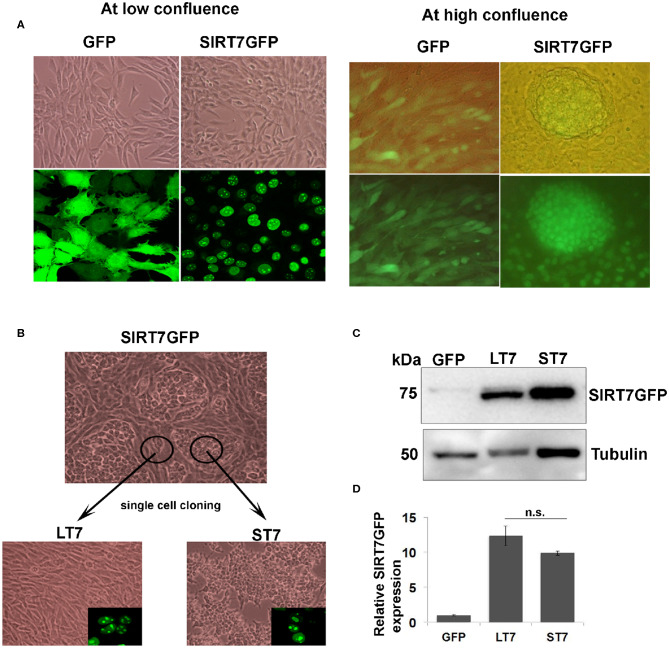
Isolation of two morphologically distinct cell lines overexpressing SIRT7. **(A)** Cellular morphology of GFP and SIRT7 overexpressing cells as seen by light and fluorescence microscopy. Top left-hand panel shows phase contrast images of GFP and SIRT7GFP overexpressing cells showing spindle cell morphology. Bottom panel shows fluorescence images of GFP with a pan cytoplasmic localization and SIRT7GFP showing nucleolar localization. The right panel shows the images of confluent GFP and SIRT7GFP overexpressing cells. Note the appearance of distinct cellular colony only in the SIRT7GFP expressing cells but not in the control GFP cells. The top panel is a merged image (bright field and fluorescence) and lower panel shows images taken only in fluorescent channel. Note, SIRT7GFP expression was seen in both the colony and the surrounding cells. **(B)** Isolation of single cell clones of SIRT7GFP overexpressing cells. Using cloning cylinders, SIRT7GFP cells were isolated both from the compact colonies and the surrounding area. The cells surrounding the colony showed long slender fibroblast morphology and were named LT7 cells. The cells inside the colonies appeared tightly adhering and small in size, hence named ST7. Both ST7 and LT7 cells show nucleolar localization of SIRT7 as revealed by confocal microscopy (inset). **(C,D)** Quantification of SIRT7GFP expression levels in LT7 and ST7 cells by immunoblotting. n.s.- non-significant.

In general, the SIRT7 expressing cells were trypisinized and re-passaged when they attained 70% confluency. Incidentally, few culture dishes which grew to confluency showed presence of distinct compact cellular colonies composed of small sized cells. These colonies were surrounded by long slender spindle shaped cells (Figure 1B). For isolation of these morphologically distinct cells, the cultures were maintained in a confluent state and the colonies were allowed to grow in size with a regular change of media. The two morphologically distinct cell types were then separated by placing cloning cylinders and trypsinizing the cells for them to detach. Following trypsinization the cells were collected by pipetting from inside the cloning cylinders and expanded by further culturing in fresh medium, followed by subsequent single cell clone isolation. The small and round cells with epithelial morphology were named ST7 (small cells overexpressing SIRT7) and those with elongated fibroblast like morphology were named LT7 (long cells overexpressing SIRT7). Both LT7 and ST7 cells were then maintained as two separate SIRT7 overexpressing cell lines with different morphologies.

### Microarray and Data Analysis

To characterize the changes associated with SIRT7GFP overexpression, gene expression profiling was performed on control GFP expressing cells and SIRT7 overexpressing LT7 and ST7 cells, using the 8X 60,000 array chips from Agilent. This array had 61,307 probes for 50,090 mouse genes which included both coding and non-coding genes. This work was done at the Genotypic facility based in Bengaluru, India.

### Data Normalization

The normalization was done using GeneSpring GX 11.5 Software. The normalization method used was Percentile shift normalization. It is a global normalization, where the locations of all the spot intensities in an array are adjusted. This normalization takes each column in an experiment independently and computes the percentile of the expression values for this array across all spots (where n has a range from 0 to 100 and *n* = 75 is the median). It subtracts this value from the expression value of each entity. Analysis was done for ST7 and LT7 cells with respect to the control cells expressing GFP alone.

### Statistical Analysis

Differential gene expression analysis was done on the normalized data. GFP expressing cells served as the reference control and the differential score was calculated for genes in LT7 and ST7 cells. A fold change greater than or equal to 0.8 in each of the replicates of LT7 and ST7, and a fold change > 1 in the geomean of the two replicates was considered up-regulated. Similarly, a fold change lesser than or equal to 0.8 in each of the replicates of LT7 and ST7 and a fold change lesser than 1 in the geomean of the two replicates were considered down-regulated. The up- and down-regulated genes were ranked on the basis of fold expression, *p*-value, and average intensity of LT7 and ST7 groups.

### Cluster Analysis

Differentially regulated genes were clustered using hierarchical clustering to identify significant gene expression patterns. The clustering algorithm used for this was “Hierarchical.” The most similar expression profiles were joined together to form a group. These were further joined in a tree structure, until all data formed a single group. Clustering was based on the Entities Linkage rule i.e., the average distance between two clusters is the average of the pair-wise distance between entities in the two clusters. Similarity was measured using the Pearson clustering algorithm which measures similarity or dissimilarity between entities.

### Immunoblotting

Cells were collected by trypsinization and lysed in ice cold NP40 lysis buffer (20 mM Tris pH 7.5, 150 mM NaCl, 1 mM EDTA, 1 mM EGTA, 1% (v/v) Nonidet P-40) containing protease inhibitor cocktail (Roche, Germany). Lysates were quantified by a BCA method and an equal amount of protein was separated on SDS-PAGE and then transferred to PVDF membranes. The blots were blocked with blocking buffer containing 5% non-fat dry milk in PBS containing 0.1% Tween20 (PBST) for 30 min, followed by incubation overnight at 4°C with primary antibodies. Detection was done using HRP-conjugated secondary antibody (Bangalore Genie, India) and developed by ECL Prime reagent (GE healthcare, USA) according to the manufacturer's protocol.

### Immunofluorescence

Cells grown on coverslips were fixed either with 4% paraformaldehyde/0.1% Triton X-100 or methanol/acetone (1: 1). It was followed by blocking with 2% BSA in NaCl/Pi for 30 min, and incubation with primary antibody in 2% BSA. After this the cells were incubated with Alexa Fluor 488- or 594-conjugated secondary (anti-mouse/rabbit) antibodies (Molecular Probes/Invitrogen, Carlsbad, CA, USA) diluted in 2% BSA. The coverslips were mounted in Vectashield mounting media containing 4, 6-diamidino-2-phenylindole (DAPI) (Molecular Probes). Imaging was done on a laser scanning confocal LSM510 microscope (Zeiss, Oberkochen, Germany) or a fluorescence inverted microscope (Olympus 1X51, Tokyo, Japan).

## Results

### SIRT7 Overexpressing Cells Induced Morphological Changes at High Confluence

SIRT7 tagged to GFP (SIRT7GFP) and a control GFP vector were stably expressed in NIH3T3 fibroblasts. SIRT7 overexpressing cells showed typical nucleolar localization while the GFP alone expressing cells showed a pan-cellular expression (Figure 1A). Previously we had reported that SIRT7 expression delayed the cell cycle progression of NIH3T3 cells (12). At low confluence there was no difference in the morphology of either GFP or SIRT7GFP overexpressing cells. It was noted that when the cells were confluent, SIRT7GFP overexpressing cells showed presence of distinct compact cellular colonies with closely adhered cells (Figure 1B), which was not observed in the confluent GFP overexpressing cells. Additionally, the cells within the tight aggregated colonies appeared smaller in size. The morphologically distinct cell colonies were surrounded by elongated spindle shaped cells with morphology typical of the parental cell line NIH3T3.

As described in the methods section, clonal populations of the two morphologically distinct SIRT7 expressing cells were then derived: (a) those with morphological transition showing conspicuously small size and growing in aggregates, which from henceforth are referred as ST7 cells (small SIRT7 cells), and (b) those which did not show any morphological transition and retained the typical spindle fibroblasts appearance, hence were larger in size and didn't show the aggregating tendency, which from henceforth are referred as LT7 cells (large SIRT7 cells). Both ST7 and LT7 showed prominent nucleolar expression of SIRT7GFP (Figure 1B). To check if the difference in morphologies was due to differential expression of SIRT7GFP, levels of SIRT7GFP were quantified by immunoblotting (Figures 1C,D). LT7 and ST7 cells had no significant difference in the expression levels of SIRT7, thereby ruling out the effect of expression levels in inducing the morphological transition. Differences in cellular architecture were highlighted by F-actin staining. Phalloidin staining revealed thick parallel bundles of cytoplasmic F-actin or actin stress fibers in vector alone GFP expressing and LT7 cells, while ST7 cells showed only membrane staining for F-actin (Figure 6D). Further, both GFP expressing and LT7 cells showed abundant cytoplasm which appeared scanty in the ST7 cells, indicating an increased nuclear to cytoplasmic ratio. In continuous culture, NIH3T3 cells can spontaneously transform into neoplastic foci, hence we tested if the ST7 cells are transformed cells by plating them on soft agar for anchorage independent growth. ST7 cells did not show any growth on soft agar (data not shown), indicating that the morphological changes associated with SIRT7 overexpression are related to cellular transition rather than cellular transformation.

### ST7 Cells Show Open Chromatin Structure With Loss of Heterochromatization

**Figure 2:**
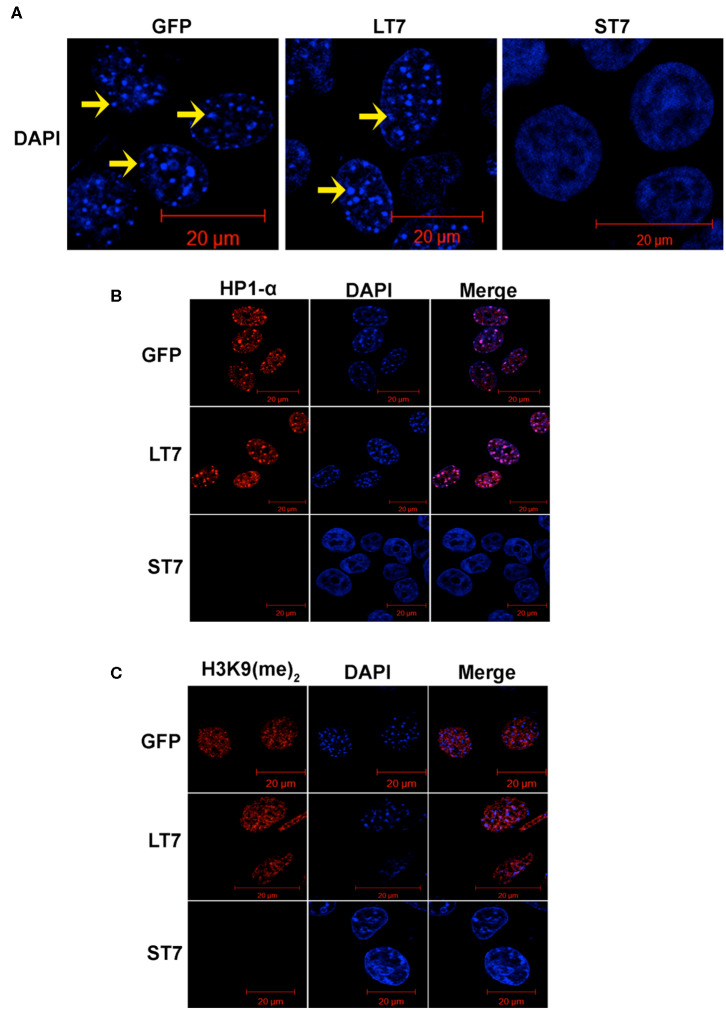
Loss of heterochromatization in ST7 cells. **(A)** Confocal images of nuclei stained with DAPI (blue). Nuclei of GFP and LT7 cells show distinct DAPI bright regions (arrow) in nucleus indicative of heterochromatic regions which is a characteristic feature of mouse cells. The nuclei of ST7 cells on the other hand displayed a conspicuous absence of DAPI bright heterochromatic foci. **(B)** Confocal images showing staining for heterochromatin protein HP1α (red). DAPI (blue) was used to counterstain the nuclei. Note the punctate pattern of HP1α corresponding to heterochromatic regions in nuclei of control GFP and LT7 cells but not in ST7 cells. **(C)** Confocal images showing staining for H3K9 dimethylation (red), an inactive heterochromatin mark. DAPI (blue) was used to counterstain the nuclei.

While performing the phalloidin staining a prominent change in the nucleus was noted between ST7 and LT7 cells. In general, the mouse nuclei show DAPI bright regions with punctate appearance, which indicate the heterochromatic structure. In accordance, prominent DAPI bright heterochromatic foci were noted in control GFP and LT7 nuclei (Figure 2A). However, the DAPI stained nuclei of ST7 cells displayed a conspicuous absence of heterochromatic foci (Figure 2A). The process of heterochromatization is marked by a protein called heterochromatization protein, or HP1, which in turn binds to methylated lysine residue of histone, H3K9 (31–33). Therefore, heterochromatization status was evaluated by both HP1 and H3K9 dimethylation status by immunofluorescence. Both control GFP cells as well as LT7 cells showed staining with HP1α and H3K9 (Me)_2_ antibodies (Figures 2B,C). Also, the HP1α staining co-localized with DAPI dense heterochromatization foci. However, ST7 cells showed absence of both HP1α staining and H3K9 di-methylation.

Heterochromatization is usually indicative of closed chromatin configuration, which is transcriptionally inactive. The absence of heterochromatin marks in ST7 cells is indicative of genome wide chromatin remodeling. Hence, a global gene expression profiling was performed to understand the changes associated with gene expression patterns following SIRT7 overexpression.

### Gene Expression Analysis of LT7 and ST7 Cells

**Figure 3:**
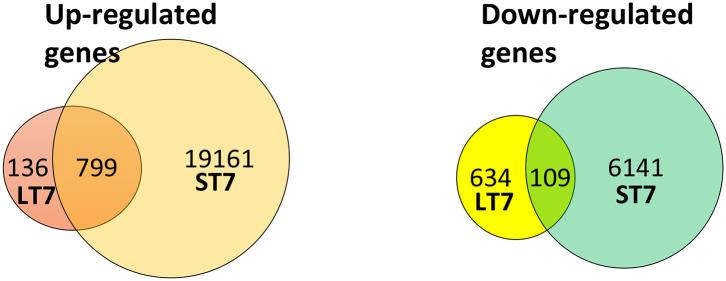
Venn diagram representing the number of differentially expressed genes in LT7 and ST7 cells. LT7 cells showed up-regulation of 935 (136 + 799) genes while ST7 showed up-regulation of 19,960 (799 + 19,161) genes. Similarly, 743 (634 + 109) genes were downregulated in LT7 cells and 6,250 (109 + 6,141) genes were downregulated in ST7 cells. A total of 1,678 (935 + 743) genes showed differential regulation in LT7 cells, while 26,210 (19,960 + 6,250) genes were deregulated in ST7 cells.

Microarray-based gene expression analysis (Agilent Technologies) was done on SIRT7 overexpressing LT7 and ST7 cells, while GFP expressing cells served as a reference control. Biological replicates (from different passages) of RNA samples from each group were analyzed. Following SIRT7 overexpression, LT7 showed differential regulation of 1,678 (5.71%) genes out of a total of 29,360 coding genes, while ST7 showed differential regulation of 26,210 genes (89.3%), indicative of a massive reprogramming (Table 1, Figure 3). As almost 90% of genes were deregulated in ST7 cells, only those genes which were deregulated in LT7 cells as a direct consequence of SIRT7 overexpression were analyzed. Hence, genes specifically altered in LT7 (1,678) were used for functional enrichment analysis.

**Table 1:** Summary of differentially regulated genes in LT7 and ST7 cells compared to control GFP expressing cells.

	**LT7**	**ST7**	**Both in**	**Either in**
			**LT7 and ST7**	**LT7 or ST7**
Up-regulated genes	935	19,960	799	20,096
Down-regulated genes	743	6,250	109	6,884
Total genes	1,678	26,210	908	

Functional enrichment analysis by DAVID for GO (Gene Ontology) revealed the differentially expressed genes belonged to varied gene families involving developmentally regulated genes, biosynthetic processes, regulation of cell adhesion, defense response etc. A list of genes involved in cellular proliferation and cell fate commitment is provided in Tables S1, S2. One of the largest class of genes that were found to be differentially regulated upon SIRT7 over-expression in LT7 were those involved in cellular adhesion. The morphological change seen in transition from LT7 to ST7 cells also indicated changes in adhesive properties of cells. A list of differentially regulated genes involved in cellular adhesion is provided in Table S3.

**Figure 4:**
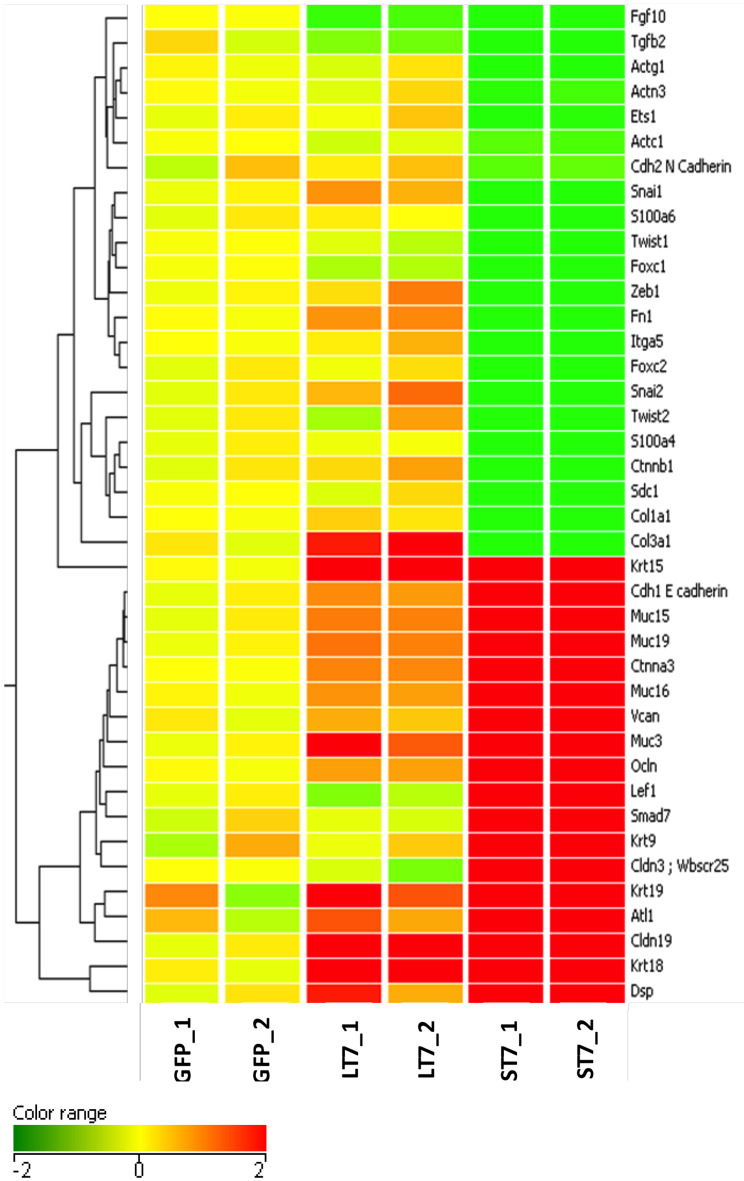
Cluster analysis of genes involved in mesenchymal to epithelial transition. A total of 40 genes responsible for maintenance of mesenchymal phenotype were clustered on the basis of the log_2_ expression ratio. Up-regulated and down-regulated genes are colored in red and green, respectively. Columns refer to the various cell lines, GFP are control cells; LT7 and ST7 are SIRT7 overexpressing cells. Note in ST7 cells the genes involved in MET are grouped in two large blocks, those which regulate epithelial characteristics are up- regulated while genes involved in mesenchymal features are down-regulated.

The morphological transition from LT7 to ST7, as well as the differential regulation of genes involved in development and cellular adhesion in LT7 cells, suggested a possible role of SIRT7 in determining cellular plasticity. The LT7 cells showed typical features of fibroblasts (mesenchymal) while the ST7 showed stubby morphology with tight adherence similar to epithelial cells. Hence, the genes involved in the EMT process were filtered and selectively clustered irrespective of their fold expression. Cluster analysis clearly showed an increased expression of genes involved in epithelialization in ST7, accompanied with down regulation of genes involved in maintenance of mesenchymal characters (Figure 4). Thus, the microarray expression analysis indicated an opposing trend of EMT thereby indicative of mesenchymal to epithelial transition (MET) during transition from LT7 to ST7.

### LT7 to ST7 Transformation Is Accompanied With N- to E- Cadherin Switch

**Figure 5:**
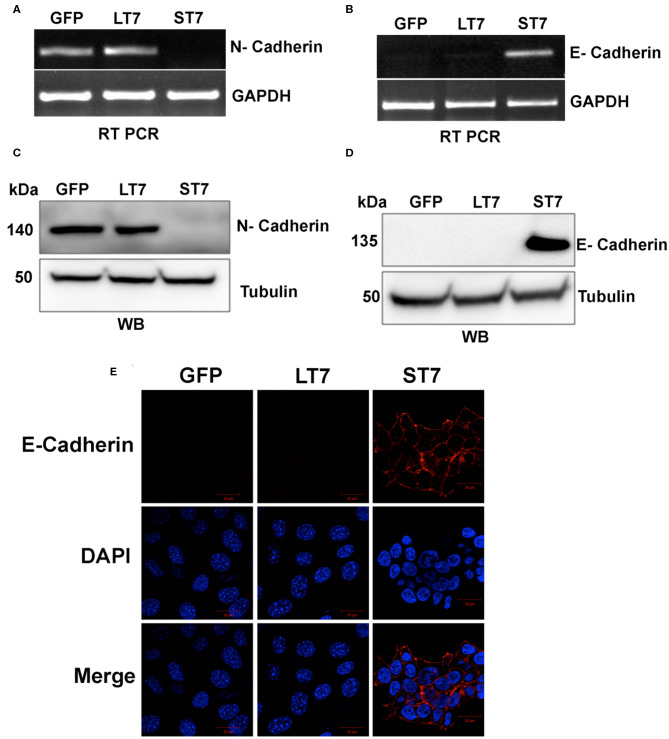
Cadherin switching in ST7 cells. **(A,B)** RT-PCR analysis showing expression levels of N- and E- cadherin. **(C,D)** Immunoblot analysis of N- and E-Cadherin expression. **(E)** Confocal images showing expression of E-cadherin (red) in ST7 cells but not in control or LT7 cells. DAPI (blue) was used to stain the nuclei.

A switch in cadherin expression is often associated with cellular plasticity during the transition from epithelial to mesenchymal state (34, 35). The global gene expression also showed changes in expression of cadherins (Table S3) following SIRT7 expression in ST7 cells. Therefore, the expression of N-cadherin and E- cadherin which serve as adhesion molecules in mesenchymal and epithelial cell types, respectively, was validated by both RT-PCR and immunoblotting assays. GFP expressing control cells as well as LT7 cells showed prominent N-cadherin expression at both transcript and protein levels, while ST7 cells showed absence of N-cadherin expression (Figures 5A,C). ST7 cells on the other hand showed expression of E-cadherin both at the transcript and protein levels, which was conspicuously absent in GFP expressing and LT7 cells (Figures 5B,D). This transactivation of E-cadherin in ST7 cells is suggestive of a gain of epithelial cell type functions. Further, the distribution of E- cadherin was checked by immunofluorescence. Only ST7 showed strong positivity for E-cadherin, which was conspicuously absent in GFP expressing as well as LT7 cells (Figure 5E). This data on cadherin switch from N- to E- type in ST7 cells is indicative of epithelialization.

### Role of SIRT7 in Induction of Adhesive Tight Junctions in NIH3T3 Fibroblasts

**Figure 6:**
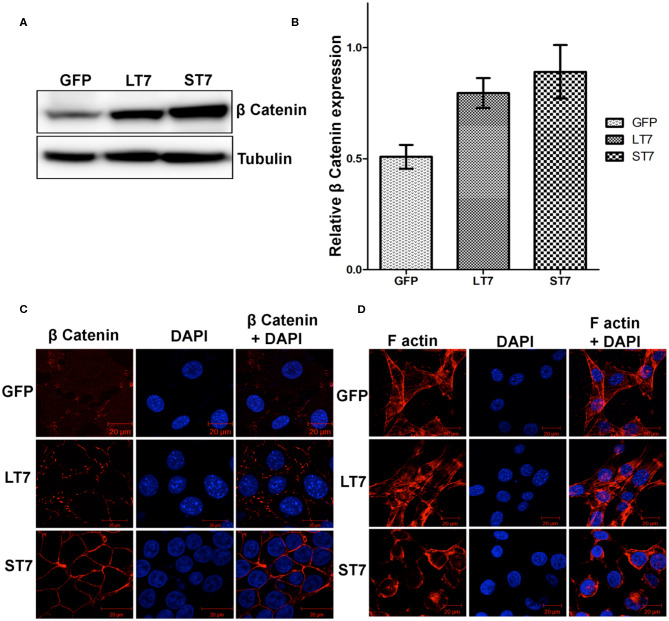
Stabilization of ß catenin following SIRT7 overexpression. **(A)** Immunoblot analysis showing a progressive increase in expression of β catenin in SIRT7 overexpressing LT7 and ST7 cells compared to control GFP cells. **(B)** Bar graph showing fold change in expression of β catenin as seen over replicate experiments as quantified by Image J. **(C)** Confocal images showing localization pattern of β catenin (red). Very feeble cytoplasmic localization is seen in GFP cells. The SIRT7 overexpressing cells show stabilization of β catenin on plasma membrane. A discontinuous punctate staining of β catenin was seen on membrane in LT7 cells while a continuous membrane staining was seen in ST7 cells. DAPI (blue) was used to counterstain nuclei. **(D)** Confocal images of cells stained with rhodamine phalloidin for visualizing the F-actin (red) which appeared as bundles of cytoplasmic stress fibers in GFP and LT7 cells, while it was redistributed to the cell membrane in ST7 cells. DAPI (blue) was used to counterstain nuclei. Note the scanty cytoplasm and prominent nucleus in ST7 cells.

As SIRT7 mediated the cadherin switch (N- to E-) in NIH3T3 cells, it was imminent to explore the expression of β catenin, which is an E-cadherin binding protein. Immunoblot analysis revealed a progressive increase in expression levels of β catenin from LT7 to ST7 cells, as compared to the control GFP expressing cells (Figures 6A,B). This suggested that SIRT7 over expression helped in stabilization of β catenin protein levels, hence we checked its localization status in LT7 and ST7 cells. Immunofluorescence-based microscopy revealed a faint cytoplasmic staining of β catenin in control GFP expressing NIH3T3 cells. However, SIRT7 overexpressing cells (LT7 and ST7) displayed an intense staining for β catenin on the cell membrane (Figure 6C). Interestingly, the pattern of β catenin distribution on the cell membrane differed between LT7 and ST7. While LT7 showed a discontinuous punctate type staining pattern for β catenin, the ST7 cells showed continuous membrane staining typical of epithelial cells (Figure 6C). These results indicated a role of SIRT7 in β catenin stabilization on the cell membrane for establishment of tight junctions. Yet another feature of tight junction in epithelial cells is membrane-bound F-actin. Immunofluorescence for F actin showed that while F-actin was predominantly associated with cytoplasmic stress fibers in both GFP and LT7 cells, it was restricted to the cell membrane in ST7 cells (Figure 6D). These results indicated a progressive stabilization of β catenin upon SIRT7 overexpression in LT7 and ST7 cells.

### SIRT7 Overexpression Down Regulates Transcriptional Regulators of Cellular Plasticity Viz., Snail, Slug and Zeb

**Figure 7:**
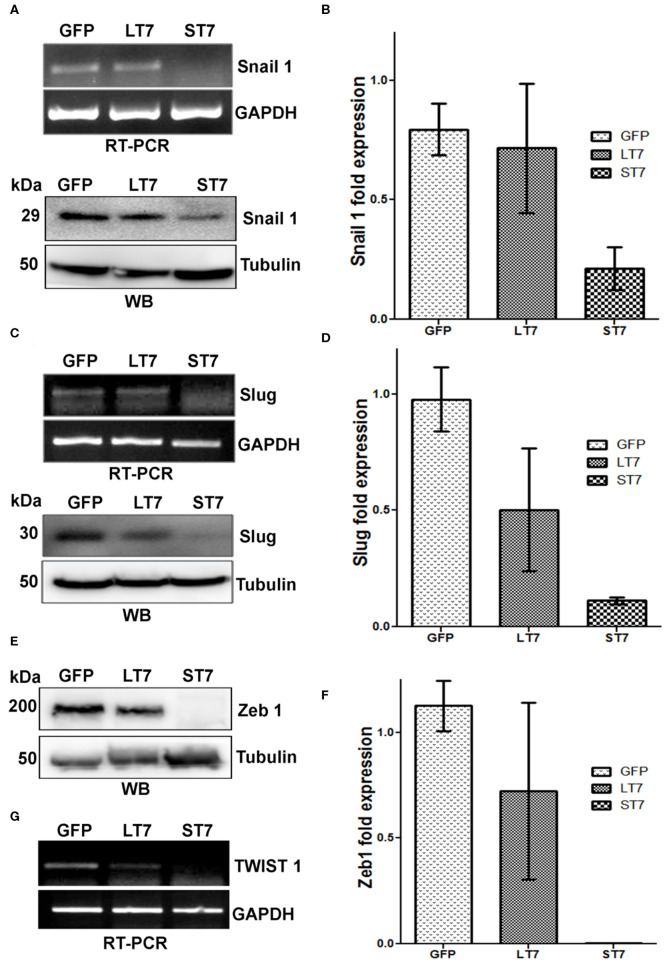
SIRT7 overexpression leads to progressive down regulation of transcriptional repressor of E-Cadherin. **(A,C)** Transcription factors Snail 1 and Slug showed a progressive decrease in transcript (RT-PCR) as well as protein levels (western blot, WB) in SIRT7 overexpressing cells (LT7 and ST7) as compared to control GFP expressing cells. **(E,G)** Western blot (WB) showing Zeb1 expression and RT-PCR for TWIST1 transcript level in control (GFP) and SIRT7 overexpressing cells (LT7 and ST7). **(B,D,F)** Bar diagram representing the fold change in the expression levels of various transcription factors at protein level over replicate experiments as quantified by image J.

The expression of E-cadherin is silenced in mesenchymal cells by the transcription factors snail and slug. Suppression of these factors is crucial for E-cadherin expression and epithelialization. In accordance with this, a progressive decline in expression levels of slug, snail and zeb1 was noted from GFP control to LT7 and ST7 cells, by both RT-PCR and immunoblot assays (Figures 7A–F). In fact, ST7 cells showed extremely feeble to almost absent expression of protein for all the three transcription factors. Absence of these transcription factors in ST7 cells alleviated the suppression on E-cadherin expression, resulting in the formation of focal adhesions and transition to epithelial-like cell state. The transcript level of TWIST1, another transcription factor involved in maintenance of mesenchymal phenotype, was also checked by reverse transcriptase PCR (RT PCR). Compared to the control GFP cells, expression of TWIST1 also showed a progressive decline in LT7 cells, with the least expression in ST7 cells (Figure 7G).

### Changes in Expression of Extracellular Matrix (ECM) Proteins

**Figure 8:**
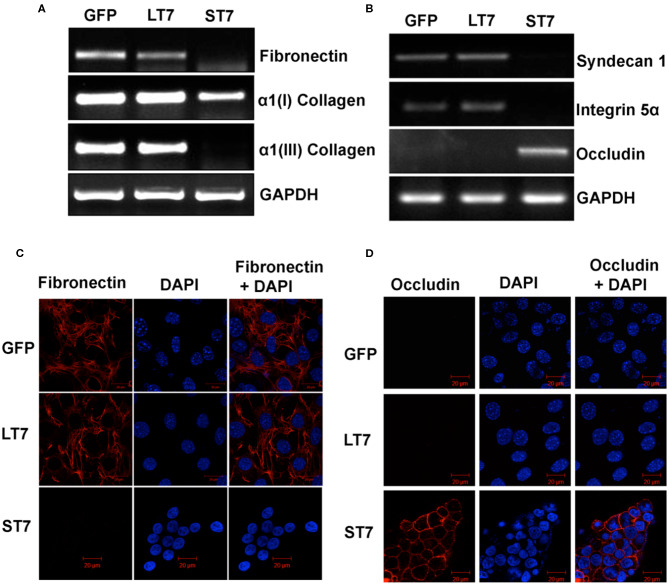
Expression levels of extracellular matrix (ECM) proteins following SIRT7 overexpression. **(A,B)** Transcript levels of Fibronectin, Collagen I, Collagen III, Syndecan I, Integrin 5α, and Occludin as evaluated by RT PCR in GFP, LT7, and ST7 cells. GAPDH served as a loading control. **(C,D)** Immunofluorescence images of GFP and SIRT7 overexpressing (LT7 and ST7) cell showing localization pattern of fibronectin and occludin.

Mesenchymal as well as epithelial cells are characterized by different profiles of extracellular matrix proteins with different repertoires of integrins, collagen, as well as laminin proteins. In view of this, the expression levels of different ECM proteins were evaluated in GFP control, LT7 and ST7 cells. The expression pattern of fibronectin was checked by reverse transcriptase PCR (RT-PCR) and immunofluorescence. Both these techniques showed the expression of fibronectin in control and LT7 cells, with its conspicuous absence in ST7 cells (Figures 8A,C). Levels of α1(I) and α1(III) collagen were checked by RT-PCR, which too demonstrated decreased expression levels in ST7 cells in comparison to both control and LT7 cells. Similarly there was a loss of Integrin 5α (Itga 5α) and Syndecan I (sdc I) at transcript levels in ST7 cells, while equivalent levels were maintained in control and LT7 cells (Figure 8B). On the other hand, ST7 cells showed expression of Occludin which was absent in GFP expressing and LT7 cells (Figure 8B). Expression of Occludin was also checked by immunofluorescence, which showed its expression only in ST7 cells (Figure 8D). Overall, the expression profile of extracellular matrix proteins showed down regulation of mesenchymal ECM proteins when SIRT7 was overexpressed, with a concomitant acquisition of epithelial ECM in ST7 cells.

## Discussion

A number of factors are involved in the decision of cell fates to transit between mesenchymal and epithelial phenotypes. This transition is accompanied with a switch of adhesive and structural proteins, which are often driven by transcription factors specific to one of these cell types. In the present work we report that SIRT7 overexpression primes mesenchymal cells for transition to epithelial phenotype. Similar to our findings, a recent report demonstrated the role of SIRT7 in promoting MET in breast cancers through SMAD4 deacetylation mediated suppression of Slug and Zeb1 transcription (26). Metastatic cancers were found to down-regulate SIRT7 to suppress this activity of SIRT7 during metastasis. Similarly in oral squamous cell carcinoma (OSCC) cells, SIRT7 is responsible for induction of E-cadherin expression and suppression of N-cadherin, Vimentin and MMP7 (27). In contrast, SIRT7 was found to promote mesenchymal transition in the context of oncogenic roles of SIRT7 in colorectal and prostate cancers, primarily by suppressing E-Cadherin expression (28–30). However, all these studies were done in epithelial cells, which have already undergone a prior process of cell transition to achieve the cancerous state. Based on these studies, there appears to be a difference in the role of SIRT7 in inducing cellular plasticity in different cell types. In our study, we used non-cancerous NIH3T3 cells to demonstrate role of SIRT7 in inducing mesenchymal to epithelial transition (MET). Against this background we could demonstrate two states toward mesenchymal transition—LT7 cells in intermediate and ST7 cells showing complete mesenchymal transition. Although only a fraction of SIRT7 overexpressing cells completed the process of epithelial transition, remaining cells (LT7) showed intermediate epithelial characters demonstrating the effect of SIRT7 on cellular plasticity. The degree of molecular changes observed in LT7 is comparable to SIRT7 reports on cellular plasticity (28–30).

Cells which failed to complete the epithelial transition had characters of an intermediate stage, as indicated by beta catenin staining and intermediary expression levels of transcription factors snail, slug and zeb1. Several recent studies have emphasized the role of intermediate staged cells in development, cancer, wound healing and fibrosis (36, 37). Nieto et al. (38) have proposed an elegant model describing intermediate stages of epithelial and mesenchymal transitions where cells can stay in multiple intermediate stages between complete epithelial and mesenchymal characters. In our report LT7 cells represent such an intermediate state of transition. Although LT7 cells did not show cadherin switch, the level of transcription factors necessary of maintaining mesenchymal characters (snail, slug, and Zeb1), was intermediate between control and ST7 cells. The most striking feature of this intermediate stage was partial and intermediate decoration of β-catenin in LT7 cells compared to a continuous staining in ST7 cells. β catenin is important in the maintenance of epithelial polarity by linking E-cadherin to α-catenin at the cell membrane for intercellular adhesion and cytoskeletal organization. Microarray expression analysis of adhesion genes in LT7 cells also showed an intermediate expression profile between control and ST7 cells.

Mouse fibroblasts have been used in a number of studies related to MET. We tried to reproduce these findings in human fibroblasts WI38 and TIG3. Unfortunately, these cell lines did not demonstrate visible transformation, probably because human cells require additional factors for such a transformation (39). Nonetheless, mouse fibroblasts have helped to delineate several pathways related to cellular plasticity (40, 41).

It has been earlier reported that SIRT7 by causing deacetylation of H3K18 (histone 3, lysine residue 18) can help in maintaining the transformed oncogenic state of cells (9). Association of chromatin-bound SIRT7 together with the SWI/SNF complex is suggestive of its role in chromatin remodeling (42). Transformation of a subset of LT7 cells to ST7 cells might be an effect of global chromatin modulation by SIRT7. This is also evident from the fact that ST7 cells showed total loss of heterochromatization, indicating complete reprogramming. However, why only a sub-population of SIRT7 overexpressing cells undergo MET while others maintained their fibroblast morphology is not fully understood. We have earlier reported that SIRT7 protects cells from DNA damage-induced senescence and apoptosis (12). In general SIRT7 is involved in mitigating several nutritional, genomic and hypoxic stresses in cells (5). While the role of SIRT7 in protection from stress is well-known, what thresholds are required to push the cells toward transitioning to an epithelial phenotype could not be addressed in this manuscript. Most probably a number of threshold barriers (along with SIRT7 overexpression and confluency) has to be crossed for a complete MET process. It is possible that some SIRT7 overexpressing cells are able to cross this barrier, thereby giving rise to two distinct populations of SIRT7 overexpressing cells. A confluent culture condition imposes constraints of space and nutritional stress. Overexpression of SIRT7 may provide a survival advantage by changing cellular fate toward an epithelial phenotype.

There are some earlier studies where the function of SIRT7 has been evaluated using the NIH3T3 cells (8, 43). However, none of these studies described the cellular transition as reported by us. This might be due to the absence of confluency in normal culture conditions, where we first observed the transition of SIRT7 overexpressing cells to epithelial morphology. Why should MET occur in conditions of confluency? While the exact mechanism is still unclear, we surmise a possible role of cellular stress, mechanical stress and nutritional deprivation in contributing toward cellular polarity and transition. Future work will be directed to delineate these components with respect to SIRT7 overexpression and cellular plasticity.

## Data Availability Statement

The original contributions presented in the study are publicly available. This data can be found here: the NCBI Gene Expression Omnibus (GSE148486).

## Author Contributions

SK and GR designed the study and wrote the manuscript together. MK did the bio-informatic analysis. All the experiments were performed by SK.

## Conflict of Interest

The authors declare that the research was conducted in the absence of any commercial or financial relationships that could be construed as a potential conflict of interest.
