# Supplementary material for: Sirtuin 7 Promotes Mesenchymal to Epithelial Transition by β-Catenin Redistribution and Stabilization
Source: Front Oncol. 2020 Jun 23;10:740. doi: 10.3389/fonc.2020.00740 (PMC7324751; doi:10.3389/fonc.2020.00740)
Supplement: Supplementary file 1 [file Data_Sheet_1.pdf]

Table S1: The subset of differentially regulated genes playing key role in regulation of cellular proliferation as revealed by DAVID analysis.

Gene	Fold_LT7_1	Fold_LT7_2	Geomean_LT7_Normalize
Agt	0.80	7.30	4.05
Bdnf	0.81	5.89	3.35
Cd24a	2.98	2.93	2.96
Cxcl10	4.39	0.91	2.65
Cyp7b1	2.32	2.58	2.45
Dbp	3.04	0.91	1.98
Dhcr7	2.30	1.56	1.93
Edn1	1.60	2.03	1.82
Efnb1	1.84	1.72	1.78
Ereg	1.29	1.68	1.49
Fabp4	1.61	1.28	1.44
Fgf10	1.29	1.53	1.41
Gata3	1.03	1.74	1.38
Gm8031	1.23	1.35	1.29
Grpr	1.49	1.09	1.29
H19	1.33	1.10	1.21
Hmx2	1.33	1.02	1.18
Id4	1.14	1.13	1.13
Igf1	1.37	0.87	1.12
Igfbp3	1.28	0.91	1.10
Ihh	1.14	1.01	1.08
Il12b	1.09	0.96	1.03
Il18	-1.06	-0.95	-1.01
Il6	-0.99	-1.07	-1.03
Jag2	-1.01	-1.06	-1.03
Lama5	-1.15	-1.00	-1.07
Lif	-1.36	-0.83	-1.10
LOC10004	-1.02	-1.23	-1.12
LOC10004	-1.39	-0.99	-1.19
Mab21l2	-1.18	-1.27	-1.23
Mitf	-1.57	-0.93	-1.25
Msx2	-1.28	-1.25	-1.26
Notch1	-1.18	-1.43	-1.30
Pax6	-1.28	-1.39	-1.33
Pmaip1	-1.36	-1.39	-1.37
Pou3f2	-1.57	-1.44	-1.50
Ptch1	-1.61	-1.70	-1.65
Ptgs1	-2.24	-1.07	-1.66
Ptgs2	-1.14	-2.42	-1.78
Slfn3	-1.99	-1.58	-1.78
Spry1	-1.91	-1.99	-1.95
Tcf3	-1.88	-2.08	-1.98
Terc	-2.33	-2.73	-2.53
Tgfb2	-2.69	-2.56	-2.63
Tgm2	-2.85	-2.51	-2.68
Tnf	-3.06	-3.07	-3.06
Vegfc	-3.48	-5.30	-4.39

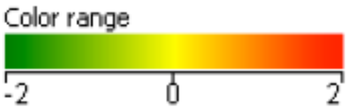

LT7_1 and LT7_2 represent biological replicates of LT7 cells.

Table S2: The subset of differentially regulated genes involved in cell fate commitment as revealed by DAVID analysis.

Gene	Fold_LT7_1	Fold_LT7_2	Geomean_LT7_Normalize
Pitx1	5.76	5.64	5.70
Hoxa11	4.92	4.92	4.92
Tbx1	3.84	4.22	4.03
Tlx3	4.67	0.95	2.81
Ptf1a	2.97	1.55	2.26
Pax6	1.60	2.03	1.82
Notch1	1.61	1.28	1.44
Gata3	1.29	1.53	1.41
Gap43	1.75	0.92	1.33
Spry1	1.23	1.35	1.29
Gfi1	1.55	0.91	1.23
Jag2	1.37	0.87	1.12
Gata2	1.29	0.92	1.11
Cyp26b1	1.22	0.95	1.08
Cdon	1.11	1.02	1.07
Tcf3	1.09	0.96	1.03
Tgfb2	-1.02	-1.23	-1.12
Notch3	-1.19	-1.06	-1.13
Fgf10	-1.36	-1.39	-1.37
Hoxd10	-1.65	-1.64	-1.65
Mitf	-2.24	-1.07	-1.66
Tal1	-1.87	-1.88	-1.87
Pax7	-3.25	-1.03	-2.14

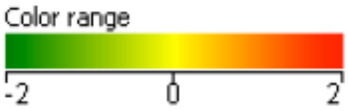

LT7_1 and LT7_2 represent biological replicates of LT7 cells.

Table S3: The subset of differentially expressed genes involved in cell adhesion as revealed by DAVID analysis.

Gene	Fold_LT7_1	Fold_LT7_2	Geomean_LT7_Normalized
Cyfip2	1.48	4.37	2.92
Amica1	2.78	2.50	2.64
Dsg4	4.19	0.86	2.53
Cldn19	2.52	2.33	2.43
Itga9	2.27	2.47	2.37
Mfap4	1.56	2.89	2.22
Edil3	1.97	2.38	2.18
Ctnnd2	3.33	0.95	2.14
Megf10	2.96	1.27	2.11
Cdh18	3.21	0.96	2.08
Pcdh9	3.15	0.89	2.02
Tnn	1.60	2.18	1.89
Olr1	2.77	0.87	1.82
Colec10	1.54	1.81	1.68
Cadm3	1.05	2.03	1.54
Postn	1.54	1.53	1.53
Fbln5	1.92	1.06	1.49
Tnfaip6	1.71	1.22	1.46
Dab1	1.89	0.88	1.39
Cntn3	1.22	1.46	1.34
Sned1	1.46	1.17	1.32
Pcdha4	1.64	0.92	1.28
Itgb2l	0.90	1.65	1.28
Lama2	0.89	1.64	1.26
LOC10004	1.20	1.31	1.26
Aebp1	1.36	1.12	1.24
Itga2	1.53	0.88	1.21
Icam5	1.28	1.08	1.18
Fat4	1.29	1.05	1.17
Adam8	1.02	1.20	1.11
Jup	0.97	1.21	1.09
Dsg1b	1.30	0.84	1.07
Nid1	1.12	1.02	1.07
Cdon	1.11	1.02	1.07
Col6a2	1.02	0.97	1.00
Mpzl2	-1.08	-0.93	-1.01
Tgfb2	-1.02	-1.23	-1.12
Myf5	-0.98	-1.33	-1.16
Col12a1	-1.02	-1.57	-1.29
Itga1	-1.45	-1.14	-1.30
LOC63458	-1.23	-1.61	-1.42
Comp	-0.82	-2.08	-1.45
Mslnl	-2.06	-1.02	-1.54
Itgbl1	-1.28	-1.81	-1.54
Vnn1	-1.82	-1.54	-1.68
Cd33	-1.84	-1.56	-1.70
Lama5	-1.14	-2.42	-1.78
Npnt	-1.98	-1.84	-1.91
LOC10004	-1.61	-2.88	-2.24
LOC10004	-1.95	-2.56	-2.26
Klra22	-1.77	-2.81	-2.29
Klra1	-1.73	-2.99	-2.36
Parvg	-2.24	-2.80	-2.52
Cd24a	-2.33	-2.73	-2.53
Thbs1	-3.46	-1.60	-2.53
Klra15	-2.89	-3.99	-3.44
Agt	-3.48	-5.30	-4.39
Cdh22	-5.30	-5.18	-5.24
Cerkl	-5.71	-5.59	-5.65

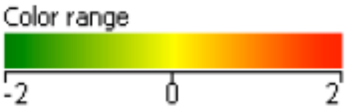

LT7_1 and LT7_2 represent biological replicates of LT7 cells.
